# The Temporal Relationship Between Ecological Pain and Life-Space Mobility in Older Adults With Knee Osteoarthritis

**DOI:** 10.1093/geroni/igaa057.2897

**Published:** 2020-12-16

**Authors:** Mamoun Mardini, Subhash Nerella, Matin Kheirkhahan, Sanjay Ranka, Roger Fillingim, Erta Cenko, Parisa Rashidi, Todd Manini

**Affiliations:** 1 University of Florida, Gainesville, Florida, United States; 2 Google, Mountain View, California, United States; 3 University of Florida, Pain Research and Intervention Center of Excellence, Gainesville, Florida, United States; 4 The University of Florida, Gainesville, Florida, United States

## Abstract

Older adults who experience pain are thought to have lower life-space mobility (spatial size and frequency of interaction with the surrounding environment). However, there is significant day-to-day variability in pain experiences that offer insights into consequences on life-space mobility that aren't understood. This study examined the temporal association between ecological pain and metrics from Global Positioning System in older adults with symptomatic knee osteoarthritis. Participants (n=19, 73.1+/- 4.8 yrs, 68.4% female) wore a smartwatch for an average period of 13.16 (+/-2.94) days. Participants were prompted in their free-living environment about their pain intensity (range 0-10) at random times in the morning, afternoon and evening. Results suggest that higher level of knee pain in older adults was associated with a 6.4 (2.81, p=0.02) fewer miles traveled per day – indicating a lower life-space mobility. A custom designed smartwatch is effective at simultaneously collecting rich information about ecological pain and life-space mobility.

